# Lanolin and Ichthyol

**Published:** 1886-10

**Authors:** 


### LANOLIN AND ICHTHYOL.

IN a paper—Notes on Drugs—in the *Journal of Cutaneous and Venereal Diseases*, Dr. H. G. Piffard says:

Lanolin, or oil from wool, comes to us from abroad in the form of a soft, foul-smelling ointment. In this country, it is protected by letters-patent No. 271,192. In the specifications, this new product is claimed to be a "perfectly white, neutral, colorless ointment," which description certainly does not apply to the article as supplied for sale.

The chief claims made in behalf of lanolin are, that it is more readily absorbed than any other fat, and that it promotes the absorption of medicinal substances combined with it. Since in the majority of cases we do not wish absorption, but surface action only, this property is a detriment, and lanolin is to be condemned as a general basis for ointments. An exception to this may be a combination of lanolin and tar, which, in suitable cases, appears to work better than the officinal tar ointment.

Ichthyol appears to me to closely resemble the old oleum sulphuratum or balsam of sulphur, and would doubtless obtain a recognized place in therapeutics, were it not that it has been, in this country at least, brought prominently forward as a general cure-all for cutaneous diseases, and advertised as such in the public journals, theatre programmes, railway stations, etc. This places it in the same rank as Cuticura and similar proprietary compounds, and removes it from the armamentarium of the scientific physician."

